# From Decades to Days: Long-Term Cognitive Functioning and Momentary Stress-Control Dynamics in Older Adults

**DOI:** 10.1093/geroni/igaf122.3593

**Published:** 2025-12-31

**Authors:** Urmimala Ghose, Oliver Schilling, Ute Kunzmann, NIlam Ram, Christiane Hoppmann, Denis Gerstorf

**Affiliations:** Humboldt University in Berlin, Humboldt University in Berlin, Berlin, Germany; Heidelberg University, Germany, Heidelberg, Baden-Wurttemberg, Germany; Universität Leipzig, Leipzig, Sachsen, Germany; Stanford University, Stanford University, California, United States; UBC, Vancouver, British Columbia, Canada; Humboldt University Berlin, Humboldt University Berlin, Berlin, Germany

## Abstract

Multiple-time scale approaches offer new opportunities to examine how developmental processes unfolding over decades shape moment-to-moment experiences in daily life. Developmental theory suggests that long-term, age-related changes provide a foundation for short-term dynamics of stress and control. Supporting this view, prior work has shown that older adults whose disease burden had increased more across the past two decades experience more daily negative affect and heightened stress reactivity. Extending this line of inquiry, we combined data from 123 old adults (65–69 years, 47% women) and 32 very old adults (85–88 years, 59% women) who provided 24+ year within-person longitudinal data on cognitive (perceptual-motor) functioning and subsequently also completed repeated daily-life assessments of control beliefs and stress six times a day over 7 consecutive days as they were going about their daily-life routines. Using Dynamic Structural Equation Models that integrate long-term growth models with location-scale multilevel models revealed that older adults with steeper cognitive decline reported lower average base levels of perceived control, more reactivity to daily stressors, and more pronounced moment-to-moment fluctuations in perceived control that were independent of stressor exposure. We take our findings to showcase developmental processes evolving over vastly different timescales are closely intertwined and discuss how such new insights will inform developmental theories of control and daily-life functioning.

